# Intensive vs. standard nurse-supervised blood pressure monitoring and intensive care utilization in acute hypertensive encephalopathy: a single-center retrospective cohort study

**DOI:** 10.3389/fmed.2026.1876586

**Published:** 2026-08-12

**Authors:** Piao Shu, Yuting Gong, Ruiling Zhu

**Affiliations:** 1 Department of Neurology, The First People's Hospital of Jiangxia District, Wuhan, Hubei, China; 2 Department of Cardiology, The First People's Hospital of Jiangxia District, Wuhan, Hubei, China

**Keywords:** blood pressure variability, hypertensive encephalopathy, intensive care utilization, nurse-led intervention, posterior reversible encephalopathy syndrome, propensity score matching

## Abstract

**Background:**

Acute hypertensive encephalopathy (AHE), a subtype of posterior reversible encephalopathy syndrome (PRES), requires rapid blood pressure control and intensive monitoring. The 2024 American Heart Association scientific statement emphasizes the need for evidence-based approaches to inpatient blood pressure management, yet the specific role of nursing surveillance intensity in AHE outcomes remains undefined.

**Objective:**

To evaluate whether intensive nurse-supervised blood pressure monitoring reduces intensive care unit (ICU) utilization and improves clinical outcomes compared with standard care in patients with AHE.

**Methods:**

This single-center retrospective cohort study included 230 consecutive adults with AHE admitted to a district general hospital in Wuhan, China (January 2022–December 2025). Patients receiving intensive nurse-supervised monitoring (blood pressure every 15–30 min, neurological assessment every 30–60 min; *n* = 119) were compared with those receiving standard care (blood pressure every 1–4 h; *n* = 111). Allocation reflected attending preference and ward/staffing availability rather than randomization or calendar period. Propensity score matching (1:1) balanced baseline characteristics. Three co-primary outcomes (NIHSS change, ICU admission, in-hospital mortality) were analyzed; because no multiplicity adjustment was prespecified, findings are interpreted as hypothesis-generating. Secondary outcomes included time to blood pressure target, blood pressure variability, and length of stay. Matched binary outcomes were compared with McNemar's test.

**Results:**

After matching, 97 pairs (194 patients) were analyzed and were evenly distributed across the four study years. The intensive monitoring group had a nominally lower ICU admission rate [74.2% vs. 86.6%; relative risk (RR), 0.86; 95% CI, 0.74–0.99; McNemar *P* = 0.038; absolute risk reduction, 12.4%; number needed to treat, 8.1], but this did not remain significant after Holm-Bonferroni correction (adjusted *P* = 0.11) or in multivariable adjustment (adjusted odds ratio, 0.51; 95% CI, 0.26–1.03; *P* = 0.061). NIHSS improvement (3.63 ± 2.49 vs. 3.36 ± 2.54; mean difference, 0.27; 95% CI, −0.46 to 1.00; *P* = 0.47) and in-hospital mortality (2.1% vs. 6.2%; RR, 0.33; *P* = 0.29) did not differ significantly. Time to blood pressure target (0.87 ± 0.58 vs. 1.20±0.77 h; *P* = 0.002) and systolic blood pressure variability (13.1 ± 2.7 vs. 15.3 ± 2.3 mmHg; *P* < 0.001) favored intensive monitoring; however, the intensive group underwent far more frequent measurement (median 28 vs. 8 readings/24 h), and these two outcomes are susceptible to measurement-frequency artifact. Hospital length of stay did not differ significantly (8.8 ± 3.9 vs. 10.4 ± 5.3 days; *P* = 0.09). Safety outcomes were comparable.

**Conclusions:**

In this retrospective cohort, intensive nurse-supervised blood pressure monitoring was associated with—but not proven to cause—a lower ICU admission rate and more efficient blood pressure control, without increased adverse events. Because the primary association was statistically fragile, susceptible to confounding by indication, and not robust to multiplicity correction, these findings are hypothesis-generating and should be confirmed in a prospective randomized trial before informing practice.

## Introduction

Posterior reversible encephalopathy syndrome (PRES), encompassing acute hypertensive encephalopathy (AHE) as its most common etiology, represents a neurological emergency requiring immediate intervention (1–3). Recent epidemiological data indicate an annual incidence of 2.7–3.1 per 100,000 individuals in the United States, with a female predominance exceeding 2:1 and a concerning upward trend attributed to improved diagnostic recognition and increasing prevalence of predisposing conditions (4). The 2025 Lancet Neurology review by Fugate and colleagues has substantially expanded our understanding of PRES pathophysiology, emphasizing the critical role of endothelial dysfunction, blood-brain barrier disruption, and the emerging significance of inflammatory cytokines including interleukin-6 and vascular endothelial growth factor (5).

The 2024 American Heart Association (AHA) scientific statement on elevated blood pressure management in acute care settings has redefined the terminology and approach to hypertensive emergencies (6). Notably, this statement eliminates the term “hypertensive urgency,” instead categorizing patients as having either “hypertensive emergency” (systolic/diastolic blood pressure ≥180/110–120 mmHg with target organ damage) or “asymptomatic markedly elevated blood pressure (7, 8).” For hypertensive emergencies including AHE, the statement recommends intensive care unit (ICU) admission with continuous blood pressure monitoring and intravenous antihypertensive therapy. However, the specific contribution of nursing surveillance intensity to achieving optimal outcomes remains inadequately defined.

Emerging evidence underscores the prognostic significance of blood pressure variability (BPV) in acute neurological conditions. A 2024 systematic review and meta-analysis by Chen et al. demonstrated that higher systolic BPV is independently associated with poor functional outcomes, mortality, early neurological deterioration, and stroke recurrence (9). Similarly, the 2024 narrative review by Zompola and colleagues emphasized that BPV independently influences functional outcomes across various stages of acute stroke care, highlighting the need for individualized approaches to blood pressure control (10–12). These findings provide a mechanistic rationale for intensive monitoring protocols that may reduce BPV through more consistent blood pressure control.

The evidence base for nurse-led interventions in blood pressure management has substantially expanded. A 2024 meta-analysis of 37 randomized controlled trials by Bulto et al. demonstrated that nurse-led interventions achieved an additional 4.66 mmHg reduction in systolic blood pressure compared with usual care (13). A complementary 2024 systematic review by Ito and colleagues confirmed both short-term and long-term efficacy of nurse-led interventions for blood pressure control (14–16). However, these studies predominantly examined chronic hypertension management in outpatient settings; evidence for nurse-led interventions in acute hypertensive emergencies, particularly in neurological critical care, remains severely limited.

The potential for intensive nursing surveillance to reduce ICU utilization carries substantial clinical and economic implications. Recent analyses indicate that ICU costs are three to five times higher than general ward care, and ICU admissions are associated with increased nosocomial complications, psychological distress, and resource constraints (17). If intensive ward-based monitoring can safely reduce ICU utilization while maintaining or improving outcomes, this would represent a paradigm shift in hypertensive emergency management with significant implications for healthcare resource allocation.

To address this critical evidence gap, we conducted a retrospective cohort study comparing intensive nurse-supervised blood pressure monitoring with standard care in patients with AHE. We employed propensity score matching to reduce confounding and hypothesized that intensive nursing surveillance would be associated with lower ICU utilization and more efficient blood pressure management without compromising patient safety. Given the observational design, our aim was to generate hypotheses rather than to establish causal efficacy.

## Methods

### Study design and ethical approval

This single-center retrospective cohort study was conducted in the Department of Neurology and Neurocritical Care of a district general hospital (The First People's Hospital of Jiangxia District, Wuhan, China) from January 1, 2022, to December 31, 2025. The institutional review board approved the study protocol (approval number: 2026K001), waiving informed consent due to the retrospective design. This manuscript adheres to the Strengthening the Reporting of Observational Studies in Epidemiology (STROBE) guidelines (18).

### Participants

Consecutive adults (age ≥18 years) with acute hypertensive encephalopathy were eligible. Inclusion criteria required: (1) acute neurological symptoms (altered consciousness, seizures, headache, or visual disturbances) within 72 h of presentation; (2) markedly elevated blood pressure (systolic ≥180 mmHg and/or diastolic ≥110 mmHg) at admission; and (3) neuroimaging consistent with PRES or exclusion of alternative diagnoses (3). Exclusion criteria included: primary ischemic or hemorrhagic stroke, traumatic brain injury, central nervous system infection, pregnancy-related hypertensive disorders, palliative care goals, hospital stay < 24 h, or incomplete outcome data.

### Exposure groups

Patients were classified based on nursing monitoring intensity during the initial 72 h. The intensive monitoring group received protocolized care: blood pressure assessment every 15 min (hours 0–2), every 30 min (hours 2–8), hourly (hours 8–24), and every 2 h thereafter; neurological assessments (NIHSS, Glasgow Coma Scale) every 30 min (hours 0–2), hourly (hours 2–8), and every 2 h thereafter; and nurse-initiated medication titration notifications based on predefined thresholds. The standard care group received conventional monitoring with blood pressure measurements every 1–4 h and neurological assessments every 4–8 h, with medication adjustments initiated solely by physician order.

### Group allocation and ICU-admission criteria

Assignment to intensive vs. standard monitoring was not randomized and was not governed by a protocol tied to a specific calendar period. In routine practice, monitoring intensity was determined by a combination of the admitting neurologist's preference and the availability of closely monitored ward beds and nursing staff at the time of admission. No institutional change mandating a transition from standard to intensive monitoring occurred during the study period, and the two groups were distributed evenly across the four study years (intensive vs. standard: 2022, 24 vs. 20; 2023, 38 vs. 33; 2024, 35 vs. 36; 2025, 22 vs. 22; χ^2^ *P* = 0.93), arguing against a secular-trend confound. Decisions to admit a patient to the ICU followed uniform institutional criteria for both groups—refractory hypertension despite intravenous therapy, declining consciousness (Glasgow Coma Scale fall ≥ 2), status epilepticus, need for mechanical ventilation, or hemodynamic instability—and were made by the treating physician independently of the nursing-monitoring assignment. We nevertheless recognize that confounding by indication from unmeasured factors influencing both allocation and prognosis cannot be excluded, and that the availability of intensive ward monitoring could in principle have lowered the threshold to manage selected patients outside the ICU.

### Outcomes

The co-primary outcomes were: (1) NIHSS change from admission to discharge (positive values indicating improvement); (2) ICU admission during hospitalization; and (3) in-hospital mortality. Secondary outcomes included: time to blood pressure target (sustained 20–25% mean arterial pressure reduction for ≥30 min); systolic and diastolic blood pressure variability (standard deviation of all measurements during 24 h); hospital and ICU length of stay; 90-day mortality; favorable functional outcome (modified Rankin Scale 0–2); and safety events [hypotension episodes (systolic < 90 mmHg), excessive blood pressure reduction (>25% mean arterial pressure decrease within 1 h), and neurological deterioration (NIHSS increase ≥4 points within 24 h)].

### Covariates

Baseline characteristics included demographics (age, sex, body mass index), vital signs (systolic/diastolic blood pressure, heart rate), neurological status (NIHSS, Glasgow Coma Scale), medical history (hypertension, diabetes mellitus, chronic kidney disease, coronary artery disease, prior stroke), clinical presentation (seizure, altered consciousness, visual disturbance), laboratory values (creatinine, estimated glomerular filtration rate, glucose), and neuroimaging findings (PRES pattern, intracranial hemorrhage, edema severity).

### Statistical analysis

Continuous variables are presented as mean±standard deviation or median (interquartile range); categorical variables as frequencies (percentages). Propensity scores were estimated using multivariable logistic regression including age, sex, body mass index, baseline blood pressure, NIHSS, Glasgow Coma Scale, diabetes, chronic kidney disease, coronary artery disease, prior stroke, and intracranial hemorrhage. One-to-one nearest-neighbor matching without replacement was performed using a caliper of 0.2 standard deviations of the logit propensity score (19, 20). Covariate balance was assessed using standardized mean differences (SMD), with SMD < 0.1 indicating adequate balance. Because seizure at presentation remained imbalanced after matching (see Results), it was additionally included as a covariate in adjusted outcome models and examined in sensitivity analyses.

Between-group comparisons used paired *t*-tests or Wilcoxon signed-rank tests for continuous outcomes and McNemar's test for matched binary outcomes; for binary outcomes, discordant pair counts informed the McNemar statistic, and relative risks (RR) with 95% confidence intervals (CI) are reported alongside for clinical interpretability. Number needed to treat (NNT) was calculated for the ICU outcome. Because three co-primary outcomes (NIHSS change, ICU admission, in-hospital mortality) were analyzed without a prespecified multiplicity adjustment and the study was not preregistered, the co-primary analyses are interpreted as hypothesis-generating, and Holm-Bonferroni–adjusted *P* values are additionally reported. Because blood pressure variability and time to blood pressure target are sensitive to measurement frequency, which differed markedly between groups, the number of blood pressure measurements per patient is reported and these outcomes are interpreted cautiously. Multivariable regression models adjusted for residual confounders, including seizure at presentation. Subgroup analyses examined effect modification by baseline NIHSS (< 5 vs. ≥5) and age (< 65 vs. ≥65 years). Sensitivity analyses included unmatched multivariable analysis, inverse probability of treatment weighting (IPTW), a seizure-adjusted model, and exclusion of patients with intracranial hemorrhage. Matching was performed in R version 4.3.0 (MatchIt, cobalt); outcome analyses were conducted in R 4.3.0 and verified in Python 3.11 (statsmodels, lifelines, scipy). Two-sided P < 0.05 indicated nominal statistical significance.

## Results

### Patient characteristics and matching

**Figure 1:**
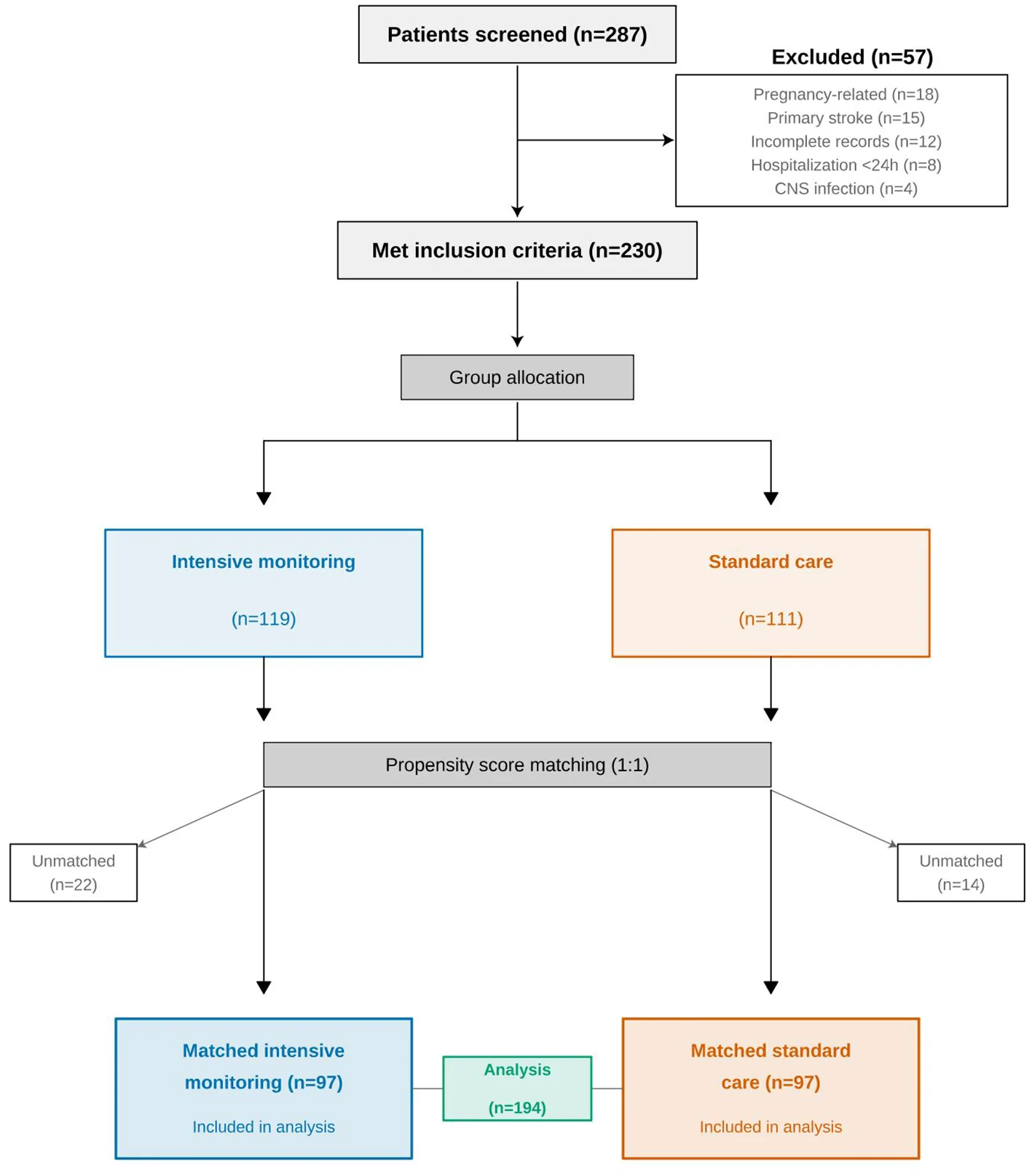
Study flow diagram. Of 287 patients screened during the study period, 230 met inclusion criteria (exclusions: pregnancy-related disorders, *n* = 18; primary stroke, *n* = 15; incomplete records, *n* = 12; hospitalization < 24 h, *n* = 8; central nervous system infection, *n* = 4). Patients were classified into intensive nurse-supervised monitoring (*n* = 119) or standard care (*n* = 111). After propensity score matching with a 1:1 ratio (caliper = 0.2 SD of logit propensity score), 97 matched pairs (194 patients) were included in the primary analysis.

Of 287 screened patients, 230 met inclusion criteria: 119 (51.7%) received intensive monitoring and 111 (48.3%) received standard care (Figure 1). Exclusions included pregnancy-related disorders (*n* = 18), primary stroke (*n* = 15), incomplete records (*n* = 12), hospitalization < 24 h (*n* = 8), and central nervous system infection (*n* = 4). The cohort had a mean age of 57.1 ± 14.1 years, female predominance (70.4%), and median baseline NIHSS of 6 (interquartile range, 4–9). Seizure was the most common presentation (66.1%), followed by impaired consciousness (65.7%).

Before matching, several imbalances existed between groups, including Glasgow Coma Scale and consciousness impairment, reflecting clinical decision-making patterns. Propensity score matching paired 97 patients per group (194 total). After matching, standardized mean differences were < 0.1 for all covariates included in the propensity model, indicating good balance; the mean propensity score was 0.512 ± 0.106 vs. 0.503 ± 0.100 (SMD 0.086). However, seizure at presentation, which was not included in the propensity model, remained imbalanced (61.9% vs. 72.2%; SMD 0.219) and was addressed through seizure-adjusted and sensitivity analyses (Table 1). The matched groups were evenly distributed across the four study years (χ^2^ *P* = 0.86).

**Table 1:** Baseline characteristics after propensity score matching.

Characteristic	Intensive monitoring (*n* = 97)	Standard care (*n* = 97)	SMD
Age, years	56.6 ± 14.1	56.7 ± 14.5	0.004
Female sex	69 (71.1%)	70 (72.2%)	0.023
Body mass index, kg/m^2^	26.5 ± 4.3	26.3 ± 3.7	0.051
Systolic BP, mmHg	175.5 ± 21.1	173.7 ± 19.1	0.089
Diastolic BP, mmHg	110.6 ± 12.5	111.7 ± 11.5	0.093
Baseline NIHSS	7.6 ± 6.0	7.5 ± 5.2	0.006
GCS score	13.1 ± 1.8	13.2 ± 1.7	0.072
Hypertension history	87 (89.7%)	86 (88.7%)	0.033
Diabetes mellitus	19 (19.6%)	22 (22.7%)	0.075
Chronic kidney disease	19 (19.6%)	20 (20.6%)	0.026
Coronary artery disease	12 (12.4%)	13 (13.4%)	0.031
Prior stroke/TIA	5 (5.2%)	4 (4.1%)	0.049
Seizure at presentation	60 (61.9%)	70 (72.2%)	0.219
PRES pattern on imaging	88 (90.7%)	90 (92.8%)	0.075
Intracranial hemorrhage	12 (12.4%)	12 (12.4%)	0.000

Values are mean ± SD or *n* (%). Standardized mean differences (SMD) were < 0.1 for all covariates included in the propensity score model. Seizure at presentation was not included in the propensity model and remained imbalanced after matching (SMD 0.219); it was addressed through seizure-adjusted and sensitivity analyses. BP, blood pressure; GCS, Glasgow Coma Scale; NIHSS, National Institutes of Health Stroke Scale; PRES, posterior reversible encephalopathy syndrome; SMD, standardized mean difference; TIA, transient ischemic attack.

### Primary outcomes

The intensive monitoring group had a lower ICU admission rate than the standard care group [72/97 (74.2%) vs. 84/97 (86.6%); RR, 0.86; 95% CI, 0.74–0.99; McNemar *P* = 0.038, based on 8 vs. 20 discordant pairs). This corresponds to an absolute risk reduction of 12.4% (95% CI, 1.3%−23.4%) and a number needed to treat of 8.1 (95% CI, 4.3–74.7). However, this nominal association did not survive Holm-Bonferroni correction for the three co-primary outcomes (adjusted *P* = 0.11) and was attenuated to non-significance in multivariable adjustment (adjusted OR, 0.51; 95% CI, 0.26–1.03; *P* = 0.061; see Sensitivity Analyses; Table 2). It should therefore be regarded as a hypothesis-generating signal rather than definitive evidence of benefit, particularly because a lower ICU admission rate may be partly entangled with the exposure itself (Discussion).

**Table 2:** Primary and secondary outcomes in the propensity score-matched cohort.

Outcome	Intensive monitoring	Standard care	Effect estimate (95% CI)	*P* value
Primary outcomes				
NIHSS improvement, points	3.63 ± 2.49	3.36 ± 2.54	MD 0.27 (−0.46, 1.00)	0.47
ICU admission	72 (74.2%)	84 (86.6%)	RR 0.86 (0.74, 0.99)	0.038^*^
In-hospital mortality	2 (2.1%)	6 (6.2%)	RR 0.33 (0.07, 1.61)	0.29
Blood pressure management				
Time to BP target, h	0.87 ± 0.58	1.20 ± 0.77	MD −0.33 (−0.53, −0.13)	0.002^*^‡
SBP variability, mmHg	13.1 ± 2.7	15.3 ± 2.3	MD −2.14 (−2.80, −1.48)	< 0.001^*^‡
DBP variability, mmHg	10.0 ± 2.6	11.6 ± 2.0	MD −1.58 (−2.24, −0.92)	< 0.001^*^‡
Healthcare utilization				
Hospital LOS, days	8.8 ± 3.9	10.4 ± 5.3	MD −1.5 (−2.9, −0.2)	0.09
ICU LOS, days†	4.0 ± 2.4	5.1 ± 3.3	MD −1.2 (−2.1, −0.3)	0.013^*^†
Functional Outcomes				
Good outcome (mRS 0–2)	70 (72.2%)	70 (72.2%)	RR 1.00 (0.84, 1.19)	0.88
90-day mortality	7 (7.2%)	12 (12.4%)	RR 0.58 (0.24, 1.42)	0.36
Safety outcomes				
Hypotension events	15 (15.5%)	13 (13.4%)	RR 1.15 (0.58, 2.29)	0.84
Excessive BP reduction	11 (11.3%)	9 (9.3%)	RR 1.22 (0.53, 2.82)	0.80
Neurological deterioration	5 (5.2%)	5 (5.2%)	RR 1.00 (0.30, 3.34)	1.00

Values are mean ± SD or *n* (%). Binary outcomes were compared with McNemar's test for matched pairs; relative risks (RR) with 95% CI are shown for interpretability. Continuous outcomes were compared with paired *t*-tests; for length of stay, the non-parametric Wilcoxon signed-rank *P* value is reported owing to skewness. †Among patients admitted to the ICU; this comparison conditions on an exposure-affected variable and is subject to collider bias. ‡Time to target and blood pressure variability are sensitive to measurement frequency, which differed between groups, and should be interpreted with caution. ^*^Nominal *P* < 0.05, not adjusted for multiple comparisons; the ICU-admission result did not survive Holm-Bonferroni correction (adjusted P=0.11). BP, blood pressure; CI, confidence interval; DBP, diastolic blood pressure; ICU, intensive care unit; LOS, length of stay; MD, mean difference; mRS, modified Rankin Scale; RR, relative risk; SBP, systolic blood pressure.

NIHSS improvement was numerically greater in the intensive monitoring group but did not differ significantly (3.63 ± 2.49 vs. 3.36 ± 2.54 points; mean difference, 0.27; 95% CI, −0.46 to 1.00; *P* = 0.47). In-hospital mortality was lower in the intensive monitoring group without reaching significance [2/97 (2.1%) vs. 6/97 (6.2%); RR, 0.33; 95% CI, 0.07–1.61; McNemar *P* = 0.29], a difference limited by the small number of events (8 deaths overall).

### Blood pressure management efficiency

**Figure 2:**
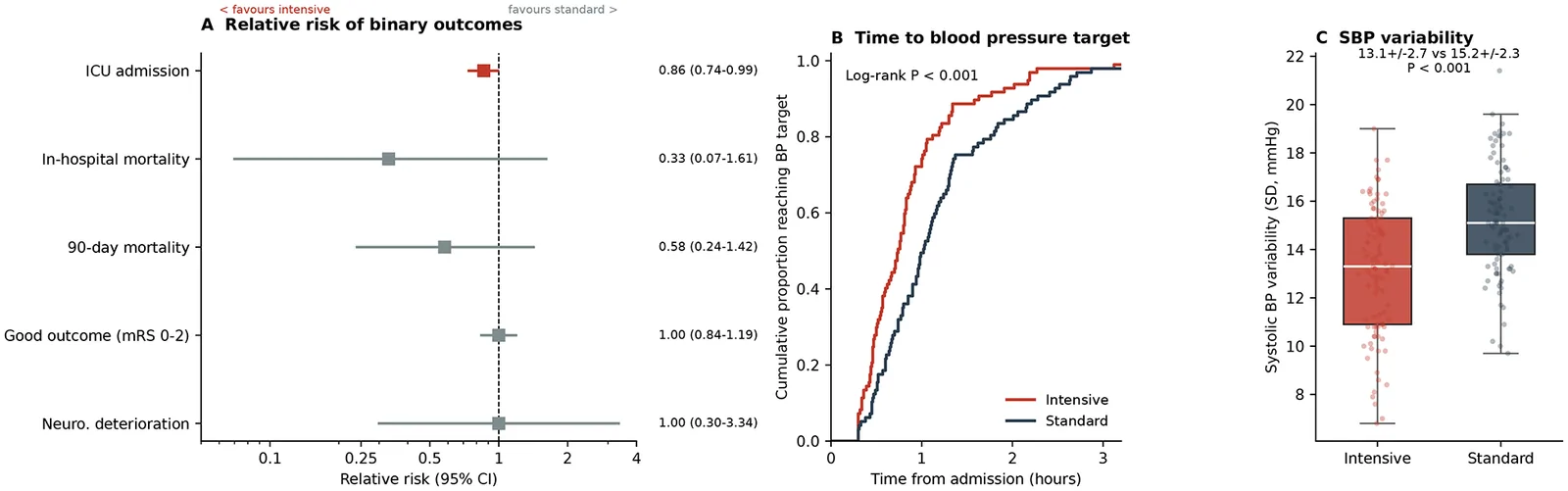
Primary and key secondary outcomes in the propensity score–matched cohort (97 pairs). **(A)** Forest plot of relative risks (95% CI) for binary outcomes; ICU admission was nominally lower with intensive monitoring (RR 0.86; 95% CI, 0.74–0.99; McNemar *P* = 0.038), although this did not survive correction for multiple comparisons. **(B)** Cumulative proportion reaching the blood pressure target over time, showing earlier recorded target achievement with intensive monitoring (log-rank *P* < 0.001); this comparison is influenced by the higher measurement frequency in the intensive group. **(C)** Systolic blood pressure variability (standard deviation of 24-h measurements; 13.1 ± 2.7 vs. 15.3 ± 2.3 mmHg; *P* < 0.001); variability estimates are sensitive to measurement frequency and should be interpreted accordingly. CI, confidence interval; ICU, intensive care unit; RR, relative risk.

The intensive monitoring group achieved blood pressure targets in a shorter recorded time (0.87 ± 0.58 vs. 1.20 ± 0.77 hours; mean difference, −0.33 hours; 95% CI, −0.53 to −0.13; *P* = 0.002), and calculated blood pressure variability was lower (systolic SD 13.1 ± 2.7 vs. 15.3 ± 2.3 mmHg, *P* < 0.001; diastolic SD 10.0 ± 2.6 vs. 11.6 ± 2.0 mmHg, *P* < 0.001). These two outcomes must be interpreted cautiously. The intensive group underwent substantially more frequent blood pressure measurement than the standard group [median 28 (IQR 24–31) vs. 8 (IQR 6–10) readings per 24 h], and across the matched cohort the number of measurements was inversely correlated with the calculated variability (*r* = −0.33, *P* < 0.001). Denser, more auto-correlated sampling tends to reduce the standard deviation, and more frequent measurement permits earlier documentation of target achievement; the apparent advantages in variability and time to target may therefore partly reflect measurement-frequency and detection effects rather than a purely biological benefit (9, 10) (Figure 2).

### Healthcare resource utilization

Hospital length of stay was numerically shorter in the intensive monitoring group, but the difference was not statistically robust (8.8 ± 3.9 vs. 10.4 ± 5.3 days; mean difference, −1.5 days; 95% CI, −2.9 to −0.2; paired *t*-test *P* = 0.024, Wilcoxon signed-rank *P* = 0.09); given the skewed distribution of length of stay, we regard this as a non-significant trend. Among the subset of patients admitted to the ICU, ICU length of stay was shorter in the intensive group (4.0 ± 2.4 vs. 5.1 ± 3.3 days; mean difference, −1.2 days; 95% CI, −2.1 to −0.3; *P* = 0.013); however, because this comparison conditions on ICU admission—an outcome itself associated with the exposure—it is subject to collider bias and is reported descriptively without causal interpretation. A formal cost-effectiveness analysis was not performed.

### Safety profile

Safety outcomes were similar between groups. Hypotension events occurred in 15.5% vs. 13.4% (*P* = 0.84), excessive blood pressure reduction in 11.3% vs. 9.3% (*P* = 0.80), and neurological deterioration in 5.2% vs. 5.2% (*P* = 1.00). Intensive monitoring was thus not associated with an excess of treatment-related adverse events.

### Multivariable regression analysis

Table 3 presents the multivariable linear regression for NIHSS change (R^2^ = 0.292). After adjustment, intensive monitoring was not significantly associated with NIHSS improvement (β = 0.27; 95% CI, −0.34 to 0.89; *P* = 0.38). Baseline NIHSS was the strongest predictor of improvement (β = 0.21 per point; *P* < 0.001), consistent with greater recovery potential in patients with more severe initial deficits. Chronic kidney disease (β = −1.19; *P* = 0.003) and intracranial hemorrhage (β = −1.33; *P* = 0.006) were independently associated with worse outcomes.

**Table 3:** Multivariable linear regression for NIHSS change (R^2^ = 0.292).

Variable	β Coefficient	95% CI	*P* value
Intensive monitoring	0.27	−0.34 to 0.89	0.38
Age, per year	−0.008	−0.030 to 0.014	0.49
Female sex	−0.24	−0.94 to 0.46	0.50
Baseline NIHSS, per point	0.21	0.15 to 0.26	< 0.001^*^
Baseline SBP, per mmHg	−0.010	−0.026 to 0.006	0.21
Diabetes mellitus	0.28	−0.48 to 1.05	0.47
Chronic kidney disease	−1.19	−1.96 to −0.42	0.003^*^
Intracranial hemorrhage	−1.33	−2.28 to −0.38	0.006^*^

Positive coefficients indicate greater NIHSS improvement. ^*^P < 0.05. CI, confidence interval; NIHSS, National Institutes of Health Stroke Scale; SBP, systolic blood pressure.

In multivariable logistic regression for ICU admission (full cohort), intensive monitoring was associated with reduced odds that did not reach significance (adjusted OR, 0.51; 95% CI, 0.26–1.03; *P* = 0.061). Baseline NIHSS was the strongest predictor (OR, 1.23 per point; 95% CI, 1.10–1.37; *P* < 0.001), consistent with severity-driven ICU utilization.

### Subgroup and sensitivity analyses

Subgroup analyses showed consistent effect directions. The NIHSS effect appeared numerically larger in patients with milder baseline deficits (NIHSS < 5: difference 0.53, *P* = 0.14; NIHSS ≥ 5: difference 0.28, *P* = 0.57), with a non-significant interaction. Effects were similar across age strata (< 65 years: difference 0.27, *P* = 0.53; ≥65 years: difference 0.22, *P* = 0.75). The direction of the ICU-admission association (lower with intensive monitoring) was consistent across all examined subgroups, although subgroup analyses were underpowered.

Sensitivity analyses indicated that the ICU-admission association was consistent in direction but borderline and method-dependent. Unmatched multivariable analysis yielded a NIHSS difference of 0.41 (*P* = 0.15) and an ICU-admission adjusted OR of 0.51 (*P* = 0.061). IPTW produced an ICU-admission OR of 0.56 (95% CI, 0.35–0.87; *P* = 0.011). A seizure-adjusted model in the matched cohort yielded an ICU-admission OR of 0.45 (95% CI, 0.21–0.99; *P* = 0.047), indicating the association was not explained by the residual seizure imbalance. Excluding the 24 matched patients with intracranial hemorrhage gave a similar estimate (OR 0.45; 95% CI, 0.19–1.04; *P* = 0.063). Across approaches the point estimate was stable (relative risk reduction ≈14%; odds ratios 0.45–0.56), but statistical significance straddled the 0.05 threshold (*P* values 0.011–0.063) and did not survive multiplicity correction; we therefore interpret the ICU-admission finding as suggestive rather than robust.

## Discussion

In this propensity score–matched retrospective cohort of patients with acute hypertensive encephalopathy, intensive nurse-supervised blood pressure monitoring was associated with a nominally lower ICU admission rate (absolute reduction 12.4%; NNT 8.1), more efficient blood pressure control, and comparable safety. We emphasize, however, that this association was statistically fragile: it did not survive correction for the three co-primary outcomes and was attenuated to non-significance in multivariable adjustment. The observational design, and in particular the uncharacterized, non-random allocation to monitoring intensity, precludes causal inference. These findings should therefore be read as hypothesis-generating evidence motivating a prospective trial, rather than as proof that intensive monitoring reduces ICU use (6).

If a genuine reduction in ICU utilization were confirmed, the implications would be substantial, because ICU care is resource-intensive [estimated daily cost $3,500–5,000 vs. $1,000–1,500 for general ward care (17)] and ICU admission carries risks of nosocomial infection, delirium, and psychological distress (21–23). Our data, however, can only suggest this possibility. A lower ICU admission rate among patients receiving intensive ward-based monitoring may also partly reflect that such monitoring functions as a partial substitute for ICU-level surveillance, making the outcome conceptually entangled with the exposure. A formal economic evaluation within a randomized design would be required to quantify any net benefit.

The observed differences in blood pressure management efficiency are biologically plausible but must be interpreted with caution. Higher systolic blood pressure variability has been associated with poor functional outcomes, mortality, and stroke recurrence (9), and reducing variability is a reasonable therapeutic goal (10). In our data, however, the intensive group was monitored approximately three to four times as often as the standard group, and both variability estimates and recorded time to target are sensitive to sampling frequency. Part of the apparent benefit in these process measures may therefore represent a measurement artifact rather than a true hemodynamic effect, and we have tempered our interpretation accordingly.

Our findings extend the established evidence for nurse-led interventions to the acute neurological care setting. While the 2024 meta-analyses by Bulto et al. (13) and Ito et al. (14) demonstrated effectiveness of nurse-led interventions in chronic hypertension management, evidence for acute settings has been lacking. A 2023 systematic review by Kappes et al. (24) on nurse-led telehealth interventions showed significant blood pressure reductions, with nurse-led approaches also improving self-efficacy and medication adherence (25, 26). Our study provides complementary evidence that nurse-led intensive surveillance in the acute, high-acuity setting can meaningfully impact both clinical and resource utilization outcomes.

The lack of statistically significant difference in NIHSS improvement warrants careful interpretation. The observed effect size (0.27 points) was smaller than the minimal clinically important difference typically cited for NIHSS (1–2 points). Several factors may explain this finding. First, the NIHSS was developed for ischemic stroke and may not optimally capture AHE manifestations, which predominantly include encephalopathy, visual disturbances, and seizures rather than focal motor deficits. Second, the relatively good baseline status of our cohort (median NIHSS 7) may have created ceiling effects limiting detectable improvement. Third, sample size limitations may have precluded detection of smaller but clinically meaningful differences. Future studies should consider AHE-specific outcome measures or composite endpoints.

Several mechanisms may contribute to the observed associations, if real. First, more frequent assessments may enable earlier detection and correction of subtherapeutic or excessive blood pressure reduction, facilitating tighter control within target ranges—relevant in AHE where ongoing hypertension drives progressive cerebral edema (27). Second, structured protocols may improve nurse-physician communication, enabling more responsive medication titration. Third, enhanced nursing presence may reduce patient anxiety and improve cooperation with care (28). Fourth, more frequent neurological assessments may enable earlier detection of deterioration. These mechanisms remain speculative in an observational design.

### Strengths and limitations

Strengths include propensity score matching with good balance on modeled confounders, multiple sensitivity analyses, use of standardized outcome measures, and inclusion of both clinical and resource-utilization outcomes. We also examined safety endpoints, confirming that the observed associations were not accompanied by increased adverse events.

This study has important limitations. First, it is retrospective and observational; allocation to intensive versus standard monitoring was non-random and determined by clinician preference and resource availability, so confounding by indication and residual unmeasured confounding cannot be excluded despite propensity score matching. Second, seizure at presentation—a defining feature of AHE—remained imbalanced after matching (SMD 0.219); although seizure-adjusted analyses were consistent, this residual imbalance is a limitation. Third, the primary ICU-admission outcome is conceptually entangled with the exposure and is statistically fragile (it did not survive multiplicity correction or multivariable adjustment), and the ICU length-of-stay analysis is subject to collider bias. Fourth, blood pressure variability and time to target are confounded by the markedly higher measurement frequency in the intensive group and may partly reflect measurement artifact. Fifth, the single-center setting at a district general hospital limits generalizability to tertiary or international centers. Sixth, the four-year window raises the possibility of temporal confounding, although the even distribution of groups across years mitigates this concern. Seventh, the study was underpowered for mortality (8 in-hospital deaths overall). Eighth, NIHSS is an imperfect outcome measure for AHE, and detailed process measures (nurse response times, titration frequency, compliance) were unavailable, precluding identification of which protocol components, if any, drove the observed associations. Finally, no formal cost-effectiveness analysis was performed.

### Clinical implications

Because this is a hypothesis-generating observational study, its findings do not by themselves justify changes to clinical practice. They do, however, identify intensive nurse-supervised monitoring as a promising, low-risk intervention worth testing prospectively, particularly where ICU capacity is constrained. The independent association of chronic kidney disease and intracranial hemorrhage with worse neurological outcomes may help identify higher-risk patients for closer observation. Any move toward formal nursing-monitoring protocols for hypertensive emergencies should await confirmatory randomized evidence.

### Future directions

Prospective randomized trials are needed to definitively establish efficacy and identify optimal monitoring frequency and protocol components. Health economic analyses incorporating direct costs, long-term outcomes, and quality of life would inform implementation decisions. Studies examining specific nursing competencies and training requirements would facilitate protocol standardization. Investigation of patient and family perspectives would inform patient-centered care models. Finally, development and validation of AHE-specific outcome measures could enhance sensitivity for detecting treatment effects in future trials.

## Conclusions

In this propensity score–matched retrospective cohort of patients with acute hypertensive encephalopathy, intensive nurse-supervised blood pressure monitoring was associated with a nominally lower ICU admission rate and more efficient blood pressure control, without increased adverse events. These associations were statistically fragile, susceptible to confounding by indication and measurement-frequency artifact, and did not survive correction for multiple comparisons; neurological improvement and mortality did not differ significantly. The findings are therefore hypothesis-generating and provide a rationale for a prospective randomized trial to determine whether structured nurse-led monitoring can safely and causally reduce ICU utilization in this high-risk population.

## Data Availability

The original contributions presented in the study are included in the article/supplementary material, further inquiries can be directed to the corresponding author.

## References

[1] Zelaya JE Al-Khoury L. Posterior Reversible Encephalopathy Syndrome. Treasure Island, FL: StatPearls (2025). 32119379

[2] Hinchey J Chaves C Appignani B Breen J Pao L Wang A . A reversible posterior leukoencephalopathy syndrome. N Engl J Med. (1996) 334:494–500. doi: 10.1056/NEJM199602223340803 8559202

[3] Fugate JE Rabinstein AA. Posterior reversible encephalopathy syndrome: clinical and radiological manifestations, pathophysiology, and outstanding questions. Lancet Neurol. (2015) 14:914–25. doi: 10.1016/S1474-4422(15)00111-8 26184985

[4] Otite FO Patel SD Anikpezie N Hoffman H Beutler T Akano EO . Demographic disparities in the incidence, clinical characteristics, and outcome of posterior reversible encephalopathy syndrome in the United States. Neurology. (2023) 101:e1554–e9. doi: 10.1212/WNL.0000000000207604 37487751 PMC10585693

[5] Fugate JE Hawkes MA Rabinstein AA. Posterior reversible encephalopathy syndrome: evolving insights in diagnosis, management, and outcomes. Lancet Neurol. (2025) 24:789–800. doi: 10.1016/S1474-4422(25)00232-7 40818477

[6] Bress AP Anderson TS Flack JM Ghazi L Hall ME Laffer CL . The Management of elevated blood pressure in the acute care setting: a scientific statement from the American heart association. Hypertension. (2024) 81:e94–e106. doi: 10.1161/HYP.0000000000000238 38804130

[7] McEvoy JW McCarthy CP Bruno RM Brouwers S Canavan MD Ceconi C . 2024 ESC guidelines for the management of elevated blood pressure and hypertension. Eur Heart J. (2024) 45:3912–4018. doi: 10.1093/ehjcvp/pvae084 39210715

[8] Lauder L Rahimi K Böhm M Mahfoud F. Debate on the 2025 guideline for the prevention, detection, evaluation, and management of high blood pressure in adults: new blood pressure targets, lower is better-and possible. Hypertension. (2025) 82:1551–8. doi: 10.1161/HYPERTENSIONAHA.125.25466 40874629

[9] Chen Y Ma Y Qin J Wei X Yang Y Yuan Y . Blood pressure variability predicts poor outcomes in acute stroke patients without thrombolysis: a systematic review and meta-analysis. J Neurol. (2024) 271:1160–9. doi: 10.1007/s00415-023-12054-w 38036920

[10] Zompola C Palaiodimou L Voumvourakis K Stefanis L Katsanos AH Sandset EC . Blood pressure variability in acute stroke: a narrative review. J Clin Med. (2024) 13:1981. doi: 10.3390/jcm13071981 38610746 PMC11012361

[11] Kamieniarz-Medrygal M Kazmierski R. Significance of pulse pressure variability in predicting functional outcome in acute ischemic stroke: a retrospective, single-center, observational cohort study. Sci Rep. (2023) 13:3618. doi: 10.1038/s41598-023-30648-2 36869131 PMC9984482

[12] Manning LS Rothwell PM Potter JF Robinson TG. Prognostic significance of short-term blood pressure variability in acute stroke: systematic review. Stroke. (2015) 46:2482–90. doi: 10.1161/STROKEAHA.115.010075 26243226

[13] Bulto LN Roseleur J Noonan S Pinero de Plaza MA Champion S Dafny HA . Effectiveness of nurse-led interventions versus usual care to manage hypertension and lifestyle behaviour: a systematic review and meta-analysis. Eur J Cardiovasc Nurs. (2024) 23:21–32. doi: 10.1093/eurjcn/zvad040 37130339

[14] Ito M Tajika A Toyomoto R Imai H Sakata M Honda Y . The short and long-term efficacy of nurse-led interventions for improving blood pressure control in people with hypertension in primary care settings: a systematic review and meta-analysis. BMC Prim Care. (2024) 25:143. doi: 10.1186/s12875-024-02380-x 38678180 PMC11056068

[15] Hwang M Chang AK. The effect of nurse-led digital health interventions on blood pressure control for people with hypertension: a systematic review and meta-analysis. J Nurs Scholarsh. (2023) 55:1020–35. doi: 10.1111/jnu.12882 36929538

[16] Clark CE Smith LF Taylor RS Campbell JL. Nurse led interventions to improve control of blood pressure in people with hypertension: systematic review and meta-analysis. BMJ. (2010) 341:c3995. doi: 10.1136/bmj.c3995 20732968 PMC2926309

[17] Dasta JF McLaughlin TP Mody SH Piech CT. Daily cost of an intensive care unit day: the contribution of mechanical ventilation. Crit Care Med. (2005) 33:1266–71. doi: 10.1097/01.CCM.0000164543.14619.00 15942342

[18] von Elm E Altman DG Egger M Pocock SJ Gøtzsche PC Vandenbroucke JP. The strengthening the reporting of observational studies in epidemiology (STROBE) statement: guidelines for reporting observational studies. J Clin Epidemiol. (2008) 61:344–9. doi: 10.1016/j.jclinepi.2007.11.008 18313558

[19] Austin PC Stuart EA. Moving towards best practice when using inverse probability of treatment weighting (IPTW) using the propensity score to estimate causal treatment effects in observational studies. Stat Med. (2015) 34:3661–79. doi: 10.1002/sim.6607 26238958 PMC4626409

[20] Vozoris NT Pequeno P Li P Austin PC Stephenson AL O'Donnell DE . Morbidity and mortality associated with prescription cannabinoid drug use in COPD. Thorax. (2021) 76:29–36. doi: 10.1136/thoraxjnl-2020-215346 32999059

[21] Needleman J Liu J Shang J Larson EL Stone PW. Association of registered nurse and nursing support staffing with inpatient hospital mortality. BMJ Qual Saf. (2020) 29:10–8. doi: 10.1136/bmjqs-2018-009219 31391314

[22] Guo X Ouyang N Sun G Zhang N Li Z Zhang X . Multifaceted intensive blood pressure control model in older and younger individuals with hypertension: a randomized clinical trial. JAMA Cardiol. (2024) 9:781–90. doi: 10.1001/jamacardio.2024.1449 38888905 PMC11195599

[23] Anderson TS Herzig SJ Jing B Boscardin WJ Fung K Marcantonio ER . Clinical outcomes of intensive inpatient blood pressure management in hospitalized older adults. JAMA Intern Med. (2023) 183:715–23. doi: 10.1001/jamainternmed.2023.1667 37252732 PMC10230372

[24] Kappes M Espinoza P Jara V Hall A. Nurse-led telehealth intervention effectiveness on reducing hypertension: a systematic review. BMC Nurs. (2023) 22:19. doi: 10.1186/s12912-022-01170-z 36650463 PMC9843665

[25] Siopis G Moschonis G Eweka E Jung J Kwasnicka D Asare BY . Effectiveness, reach, uptake, and feasibility of digital health interventions for adults with hypertension: a systematic review and meta-analysis of randomised controlled trials. Lancet Digit Health. (2023) 5:e144–e59. doi: 10.1016/S2589-7500(23)00002-X 36828607

[26] Zhang W Mei Z Feng Z Li B. Nurse-led digital health program for home blood pressure monitoring in stroke patients: protocol for a pooled analysis of randomized controlled trials. Front Public Health. (2024) 12:1378144. doi: 10.3389/fpubh.2024.1378144 39104894 PMC11298470

[27] Anderson CS Huang Y Lindley RI Chen X Arima H Chen G . Intensive blood pressure reduction with intravenous thrombolysis therapy for acute ischaemic stroke (ENCHANTED): an international, randomised, open-label, blinded-endpoint, phase 3 trial. Lancet. (2019) 393:877–88. doi: 10.1016/S0140-6736(19)30038-8 30739745

[28] Genna C Thekkan KR Raymakers-Janssen P Gawronski O. Is nurse staffing associated with critical deterioration events on acute and critical care pediatric wards? A literature review. Eur J Pediatr. (2023) 182:1755–70. doi: 10.1007/s00431-022-04803-2 36763191

